# Boolean network simulations for life scientists

**DOI:** 10.1186/1751-0473-3-16

**Published:** 2008-11-14

**Authors:** István Albert, Juilee Thakar, Song Li, Ranran Zhang, Réka Albert

**Affiliations:** 1 Huck Institutes for the Life Sciences, Pennsylvania State University, University Park, Pennsylvania, USA; 2 Physics Department, Pennsylvania State University, University Park, Pennsylvania, USA; 3 Biology Department, Pennsylvania State University, University Park, Pennsylvania, USA; 4 College of Medicine, Hershey, Pennsylvania, USA

## Abstract

Modern life sciences research increasingly relies on computational solutions, from large scale data analyses to theoretical modeling. Within the theoretical models Boolean networks occupy an increasing role as they are eminently suited at mapping biological observations and hypotheses into a mathematical formalism. The conceptual underpinnings of Boolean modeling are very accessible even without a background in quantitative sciences, yet it allows life scientists to describe and explore a wide range of surprisingly complex phenomena. In this paper we provide a clear overview of the concepts used in Boolean simulations, present a software library that can perform these simulations based on simple text inputs and give three case studies. The large scale simulations in these case studies demonstrate the Boolean paradigms and their applicability as well as the advanced features and complex use cases that our software package allows. Our software is distributed via a liberal Open Source license and is freely accessible from

## Introduction

At the most general level systems biology approaches consist of two steps. The first is building a *model *of the biological system of interest, a representation that incorporates existing knowledge and experimental observations. This model then can be subjected to various conditions and may be allowed to evolve in time, a step typically referred to as *simulation*. These simulations then can be used to generate qualitative or quantitative predictions on the overall behavior of the system.

Mathematical models of biological systems range from continuous to discrete (based on the representation of the status of the system's components) and from deterministic to stochastic (based on their incorporation of randomness and noise) [1-4]. The simplest models assume that each element of the system has a binary (Boolean) state, and are therefore discrete, deterministic and parameter-free. However, several extensions of the Boolean modeling formalism also allow for iterative parameterization and the incorporation of continuous and stochastic elements [5-7]. The most important barrier precluding a more general use of Boolean models consists of the difficulties of the computational implementation of a given model. This implementation needs to perform in a consistent, correct and extensible manner to allow for the integration of all empirical knowledge as well as the unfettered exploration of the dynamical behaviors allowed by the model. There are very few tools that focus on qualitative modeling, for example the Genetic Network Analyzer [8] supports qualitative predictions via a piece-wise linear model and provides advanced visualization capabilities. There are several software packages that focus on quantitative modeling via differential equations [9-11], and discrete modeling [7,12] but these tools are less suited for exploratory analyses of biological systems in which the majority of kinetic parameters are unknown.

In this paper we present a software toolbox (from now on referred to as BooleanNet) that greatly facilitates the implementation and study of Boolean dynamic models of biological systems. It is a tool that can simulate a Boolean model based on a very simple text based input describing the interactions and regulatory relationships in the system. The main distinguishing feature of our software compared to previous efforts is that we aim to provide support for modeling the dynamic behavior of well defined biological sub-systems, rather than focusing on a larger scale network inference, analysis or modeling based on high throughput data. Once the rules are expressed the software can employ several simulation strategies: synchronous iterations, stochastic updates or hybrid modeling via a system of piecewise linear differential equations. More importantly our system allows the integration of non-boolean mechanisms into the simulation thus expanding its applicability to a wider domain. Every aspect of the simulation process may be customized: node states may be overridden at different stages of the operation, updating rules may be altered, and differential equations may be augmented or replaced. We place particular focus on the documentation and demonstrate use cases from simple usage samples to advanced and realistic examples. A series of 6 tutorials guide users through various aspects of the simulations. Our code, written in the Python programming language, is available as a software library and is distributed via a liberal Open Source license, freely accessible through the Google project hosting service at 

### Boolean network models

A Boolean network model is a directed graph (network) whose nodes represent the elements of a system, edges represent regulatory relationships between elements, and every node is characterized by a True (1) or False (0) state [12-14]. Thus a network with N nodes will have 2^N ^possible states. As time passes the state of each node is determined by the states of its neighbors (nodes that point to it), through a rule called a **transfer function**. In the simplest case the transfer function can be represented as a statement acting on the inputs via a logical function using the logical operators **NOT**, **AND**, **OR**; this statement also returns a True/False state. Depending on the output of the transfer function, the state of the node either stays the same or changes. A change in the state of a given node generally triggers changes in the state of nodes regulated by it (the nodes it points to). This way the state of the network goes through a dynamic trajectory.

This type of Boolean representation is very common in biology, although hardly if ever is named as so. For example in the case of a gene regulatory network scientists routinely describe genes and pathways as being activated or repressed (On/Off), the analysis of gene expression levels leads to a classification of genes as being differentially expressed or not, during an infection a pathogen is either cleared or it persists. This classification is a very natural desire to order and categorize observed phenomena, and intuitively estimate the outcome of regulatory mechanisms. However, the complexity of biological networks makes even the enumeration of network components and interactions a daunting task, and human intuition must be complemented by formal modeling. Boolean models and their extensions (collectively called qualitative models) have the common feature of very closely reflecting the topology of the regulatory network (i.e. the upstream/downstream regulatory relationships invoked in verbal pathway descriptions). In addition they readily incorporate inhibitory relationships (through the NOT operator) and combinatorial regulation of a target node by several regulators, whether these regulators act independently (represented by the OR operator) or they are conditionally dependent (represented by the AND operator). Different types of qualitative models offer a range of choices for representing the passing of time, from regular time steps to sampling over relative timescales and to explicit incorporation of known decay times.

In its simplest formalism a Boolean model assumes that the processes represented as edges in the network have similar durations, and correspondingly node states are updated at multiples of a fixed time step [15]. In our software we call this mode of operation as **synchronous update**. During each iteration nodes are updated to their new values only after all rules have been applied, thus the order of updates does not affect the outcome. Synchronous Boolean models starting with a given initial condition will always reach the same state after the same number of steps, i.e. the system is entirely deterministic. Moreover, due to the finite number of states attainable by the system, the system's trajectory in state space will ultimately converge into an attractor, either a single state called a **steady state **or a repeating sequence of states called a **cycle **and characterized by a cycle length. Any given Boolean model has one or several attractors, and each attractor is associated with a set of states (called its basin of attraction) that if used as an initial condition, converge into that attractor. In synchronous Boolean models the basins of attraction of each attractor are non-overlapping. Even in this simple case much can be learned from varying the initial conditions and analyzing the model's steady states and cycles (or lack thereof). We may be able to indentify nodes (variables) that are more important and drive the early appearance of steady states, or we may find nodes whose effect matters very little, either because of the redundancy of the pathway topology or because their effect is being cancelled out. Our software implements a steady state and cycle detection routine that can be used to quickly determine their length and first occurrence.

The synchronous model cannot properly account for the different time scales over which various events take place in the biological system. Most often these time scales are not known, nonetheless imposing the equality of all time scales, as the synchronous model does, introduces an artificial constraint. We can extend the base model to account for timescales by allowing the node updates to affect the state instantaneously while performing the updates in a random order within each iteration [5,2]. This extension introduces stochasticity into the evolution of the system and allows it to sample all timescales. In this mode of operation (named **asynchronous update **in BooleanNet) the study of the network most often consists of performing a large number of replicate simulations that start from the same initial condition, then tallying the attractors reachable from this initial condition (there can be several due to the stochasticity of the asynchronous update) and the states of the nodes at different time steps. This model can be further expanded by introducing rank ordered updates, essentially grouping nodes into separate ranks based on information regarding the relative duration of their synthesis or decay. The update orders in each rank group are randomized but are executed before the rules contained in a higher rank group. This mode is also supported by our software, where the ranks are numbers affixed to each rule, and rules sharing a certain number are considered a rank group.

The piece-wise linear formalism allows us to build specialized Boolean rules that can represent each individual node in even greater detail [16]. This model associates two variables to each node: a continuous variable akin to a concentration, and a discrete variable akin to the activity of the node. The change in the continuous variable corresponding to a given node is determined by a differential equation that combines a synthesis term given by a Boolean function of the activities of the nodes regulating the node and a free (uncatalized) degradation term. The discrete and continuous variables are connected via two parameters: a decay rate for the continuous variable and a threshold that determines the minimum concentration for which the discrete variable is True. Thus a Boolean rule of the form ***A ***= ***B ****AND ****C ***will be replaced by a differential equation of the form:

$$\frac{d\;conc_{A}}{dt}=\left(\left(conc_{B}>threshold_{B}\right)and\left(conc_{C}>threshold_{C}\right)\right)-decay_{A}\times conc_{A}$$

Above an inequality will numerically evaluate to 1 if the statement is True and 0 otherwise. Here the Boolean (first) term corresponds to the *regulated synthesis *of A while the decay (second) term corresponds to its *free (unregulated) dissociation*. When the Boolean term is *True *the equation is of the form $\frac{d\;conc_{A}}{dt}=1-decay_{A}\times conc_{A}$, leading to an asymptotic increase to *1*/*delay*_*A*_, or maintenance of an initial concentration equal to this value. When the Boolean term is *False *the equation is of the form $\frac{d\;conc_{A}}{dt}=-decay_{A}\times conc_{A}$, leading to an exponential decrease to zero, or maintenance of an initial concentration equal to zero. The limiting values 0 and *1*/*delay*_*A *_of the continuous variable represent, respectively, the absence of species *A *and maximal concentration of species *A*, and correspond to the discrete values False (0) and True (1). Note that the steady states of a Boolean model are the same, regardless of the mechanism of update, and coincide with the steady state of the Boolean variables of the corresponding piecewise linear model.

## Case studies

Below we present a number of realistic scenarios that apply the BooleanNet library to several different problem domains. The rule sets, data and the code that produce the plots are part of the software package and can be found in the 'examples' directory. The case studies are based on published models; our main goal for presenting these is to demonstrate the modeling aspect of the approaches, to illustrate and explain customizations and adaptations that are often necessary to capture the biological phenomena of interest. Biological modeling is rarely "pure" in the sense of being able to fully and rigorously adhere to the mathematical formalisms. There are always elements, actors and processes do not completely fit the model, and need to be altered to accommodate some observed behaviors or limits. We believe that our software allows for an unprecedented customization, where all aspects of the simulation process can be interacted with, nodes, rules and states can be observed and modified in every single timestep. The rules, simulation and visualization code for each of the examples below can be found in the main source code distribution. Each of the three projects below have been published as separate article [18-20] that should be consulted for a more in depth description on the details of the biological systems and of the dynamic models. On our website we also distribute a series of accessible tutorials that gradually introduce the concepts used in the simulations.

### Case study 1: Modeling abscisic acid (ABA) – induced stomatal closure in plants

Plants take up carbon dioxide for photosynthesis through microscopic pores called stomata. The size of the pores is determined by the two cells (called guard cells) that flank the stomata, and the shape of the guard cells is in turn determined by their water content and turgor pressure. Plants also lose water by transpiration through the pores, thus they need to regulate the size of the stomata to balance carbon gain with water loss. During drought conditions plants synthesize a hormone called abscisic acid (ABA). ABA acts as a signal to a complex signal transduction pathway leading to the closure of the stomata. Following an extensive curation of the experimental literature, in [17] we have synthesized a network for ABA-induced closure that includes 43 nodes and 69 regulatory edges (see website or source distribution for the rules). The nodes of the network include proteins, ion channels and secondary messengers, as well as more abstract concepts such as membrane depolarization and stomatal closure. The state of 38 of the 43 nodes is regulated by other nodes in the network and in [17] we expressed this regulation as Boolean rules. Here we use these rules to compare the asynchronous and piece-wise linear frameworks of BooleanNet in exploring the dynamics of the system.

**Figure 1:**
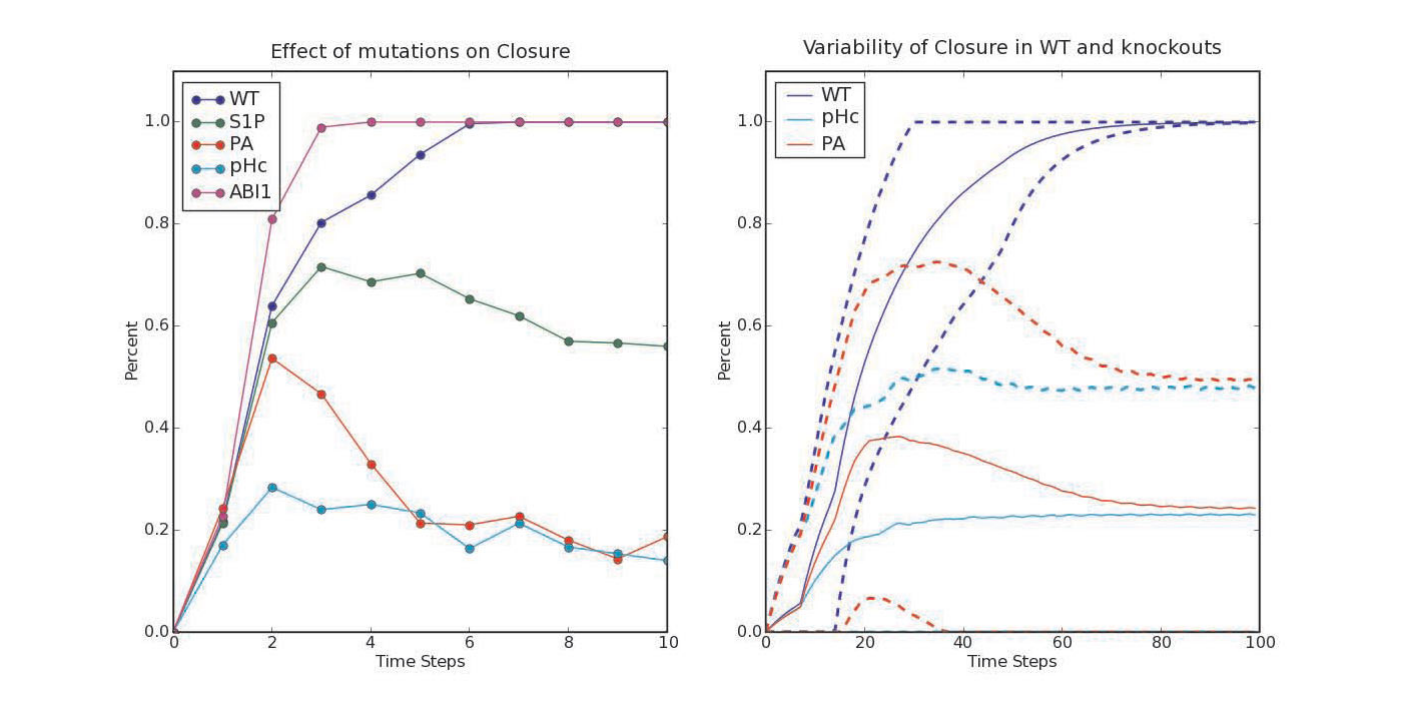
**Abscisic Acid signaling simulations: a) Effect of mutations on closure in asynchronous Boolean simulation of ABA induced closure.** Blue line indicates the closure response  (percentage of simulations with Closure = 1) in wild type (WT). Three  other knockout mutants: S1P (green line), PA (red line) and pH_c_  (light-blue line) were shown experimentally to be less sensitive to  ABA in term of ABA-induced stomatal closure. b) Variability of closure  in WT, pH_c_ knockout and PA knockouts in a piece-wise linear  simulation. The mean of the continuous variable corresponding to the  node Closure in 300 simulations is plotted as a solid line and dashed  lines indicate the mean plus or minus standard deviation. In the WT  simulation the variation of closure (blue lines) is small during the  first 20 time steps, increases from 20 to 50 time steps and gradually  decreases to 0 after 50 time steps. In PA (red lines) and pH_c_ (light  blue lines) knockout mutants, although the mean closure responses are  similar to that of WT, the variances are not decreasing after 50 time  steps.

We performed an asynchronous Boolean simulation using 300 random initial states, with ABA on for all 10 steps of the simulation. In the piecewise linear mode we set the decay rates of all nodes equal to 1 and each threshold *θ *equal to 0.5, and we performed 300 simulations with random initial states. For both modes, we tested the wild type as well as a number of important knockouts and plotted the average state of the node "Closure" as a function of time steps. Specifically, in the asynchronous Boolean simulation we plot the percentage of simulations that have Closure = 1, while in the piecewise linear simulation we plot the mean and mean ± standard deviation of the continuous variable corresponding to closure. In the wild type (WT) simulation, all nodes are updated as specified by the Boolean rules. We also performed knockout simulations of sphingosine one-phosphate (S1P), phosphatidic acid (PA), cytosolic pH (pH_c_) and abscisic acid insensitive (ABI1), setting the corresponding node's state to False (0). We found that for WT simulations, the two model variants give the same closure response (ABA Figure 1a/b, blue curves). In addition, the two simulations produced qualitatively similar results for knockouts. We also observed that the variation of the closure response was smaller in WT than in the mutants, whereas the mutants showed similar, higher than WT variations. These results suggest that knockouts not only affect the mean closure responses but also their variation [17].

### Case study 2: T-cell large granular lymphocyte leukemia simulation

**Figure 2:**
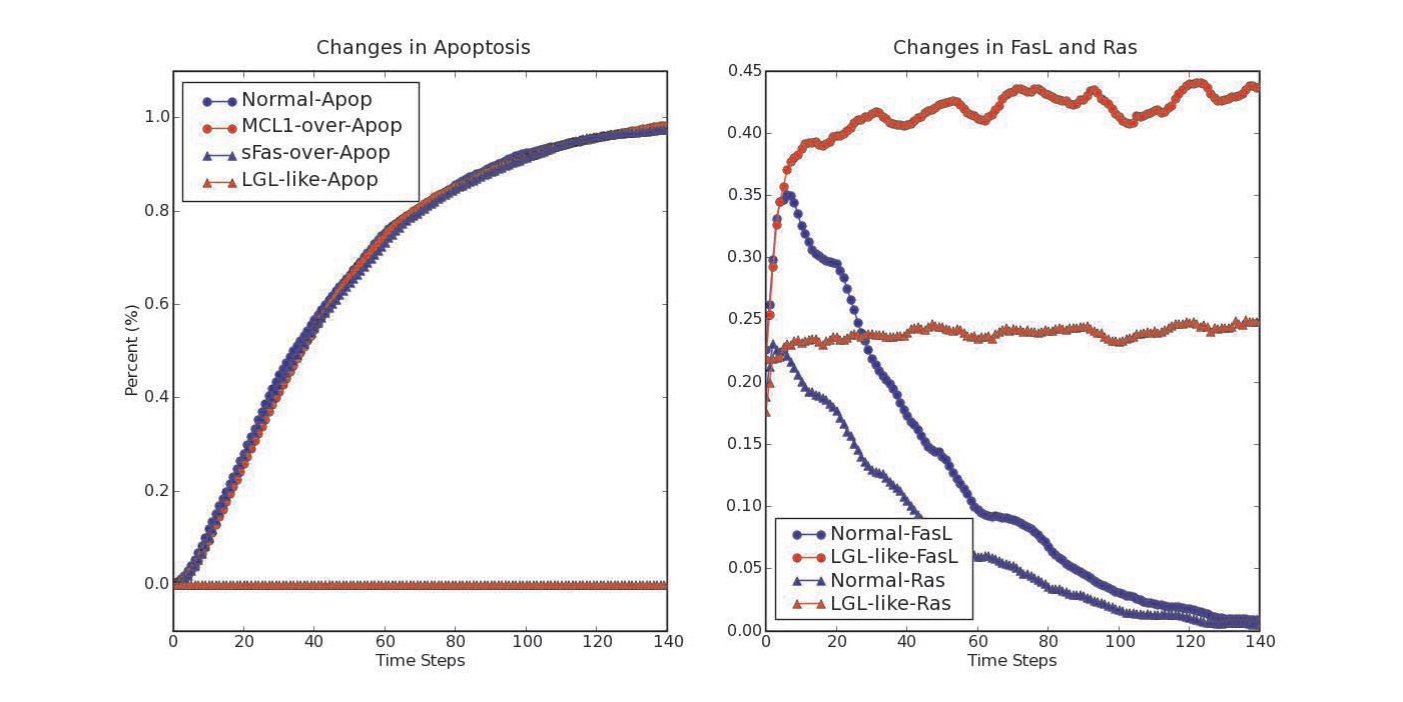
**Representative outputs of simulating the long-term survival of leukemic T-LGL cells.** (a). Inhibition of AICD by constitutive overexpression of MCL1 and sFas. Chronic antigen stimulation will induce the depletion of reactive CTL through AICD, as suggested by the asymptotic increase to 1 of apoptosis percentage in the output "Normal-Apop". Constitutive overexpression of MCL1 or sFas alone does not rescue reactive CTL from AICD, as suggested by the output "MCL1-Apop" and "sFas-Apop". However, when simulating the simultaneous overexpression of MCL1 and sFas, resistance to AICD was achieved, see output "LGL-like-Apop", as observed in leukemic T-LGL cells. (b). Additional characterization of the effect of simultaneous overexpression of MCL1 and sFas. In addition to the inhibition of AICD, simulations under conditions mimicking the constitutive overexpression of MCL1 and sFas also reproduced the known deregulated signaling pathway components in leukemic T-LGL cells, such as constitutively overexpressed FasL ("LGL-like-FasL") and constitutively activated Ras ("LGL-like-Ras"), were reproduced compared to simulations mimicking normal CTL activation ("Normal-FasL" and "Normal-Ras").

T-cell large granular lymphocyte leukemia (T-LGL) is characterized by the abnormal clonal expansion of antigen-primed mature cytotoxic T lymphocytes (CTLs) potentially driven by chronic virus infection [18]. Different from normal CTLs which are eliminated through activation induced cell death (AICD) after antigen encounter [19], leukemic LGL cells persist in the peripheral blood and remain long-term competent [18]. Fas-induced apoptosis is crucial for normal AICD [19]. Despite the high-level expression of both Fas receptor and Fas ligand (FasL), leukemic T-LGL cells are resistant to Fas-induced apoptosis [20]. Leukemic T-LGL cells express high-level of soluble Fas (sFas) which serves as decoy Fas receptor to inhibit normal Fas-induced apoptosis. In addition, it has been shown that the pro-survival MAPK pathway [21,22] and JAK-STAT pathway [23] are constitutively active in leukemic T-LGL, and anti-apoptotic BCL2 (B-cell leukemia/lymphoma 2) family member MCL1 (myeloid cell leukemia sequence 1) is overexpressed [23]. In order to examine the effect of these known deregulations on normal AICD process, in [24] we constructed the AICD network representing the main events occurring during normal CTL activation and AICD process from literature. Proteins, mRNAs and small molecules (such as lipids) are represented as nodes in the network. "Apoptosis" is also included as a node to summarize the biological effect. We used "Stimuli" as a general node indicating antigen stimulation. Interactions or regulations between nodes were represented by edges, starting from the upstream regulators and ending at the downstream targets. Here we consider a simplified version of the network and the corresponding Boolean rules in a simulation using the asynchronous mode of BooleanNet (see website for the rules). Except nodes known to have basal level activities, most of the nodes in the network were initiated in the state of 0 to reproduce a resting-T-cell-like state. We then explored the dynamics under chronic antigen stimulation via constantly setting node "Stimuli" at the state 1. This condition induces the depletion of reactive CTL through AICD, as illustrated by the asymptotic increase to 1 of the apoptosis percentage in the output "Normal-Apop" of Figure 2a. Overexpression of MCL1 or sFas alone by constantly setting the node "MCL1" or "sFas" in the state 1 does not prevent the onset of apoptosis. However, simultaneous overexpression of MCL1 and sFas completely inhibits the apoptosis onset. At the same time, Figure 2b shows that the oeverexpression of MCL1 and sFas induces the constitutive activity of Ras (a key component of the MAPK pathway [21]) as well as the constitutive expression of FasL, reproducing an LGL-like state This example illustrates that complex biological behaviors such as apoptosis can be successfully modeled using a simple Boolean model (see [24] for further results).

### Case study 3: Modeling the mammalian immune response to B. bronchiseptica infection

The dynamic interplay between a pathogen (e.g. virus or bacterium) and its host's defenses decides whether the pathogen will be cleared or will establish a niche in the host. The gram-negative bacterium *Bordetella bronchiseptica *persists within its mammalian hosts by interfering with the hosts' immune responses [25]. In [26] we synthesized a network of interactions among the immune effectors of a mammalian host and the virulence factors of *B. bronchiseptica *from the experimental literature. The network contains 34 nodes (see website for rules) that represent immune cell types, signaling molecules (cytokines), antibodies and bacterial factors such as antigens. The regulatory edges of the network represent immune processes such as antigen presentation, activation of immune cells as well as the modulation of immune components by bacteria. Based on our best interpretation of the experimental literature we formulated a piece-wise linear model and we used BooleanNet to simulate the interplay between host immune components and bacterial factors.

The piece-wise formulation was replaced by specific ordinary differential equation for the nodes 'bacteria' and 'phagocytosis' to incorporate known details of dynamic behavior. We implemented spatial separation of certain cells and cytokines by defining distinct and linked compartments. Intercompartmental transport of cells was given by Hill type functions, e.g.

$$\frac{d\;DC_{2}}{dt}=\frac{DC_{1}^{n}}{DC_{1}^{n}+H^{n}}-\gamma_{DC}\;DC_{2},$$

where *DC *represents the concentration of dendritic cells and subscripts 1 and 2 indicate the concentrations in different compartments. Since cytokines flow to various places through lymph and blood their inter-compartmental dynamics were given by the random variables *rc *and *r *so that a fraction *rc *- *r *<*f *<*rc *+ *r *of the total concentration of the cytokine is present in the compartment in which the cytokine is produced and a fraction *1*- *f *is transported to the other compartment. We changed the fraction *f *after a variable expiration time several times during the simulation.

**Figure 3:**
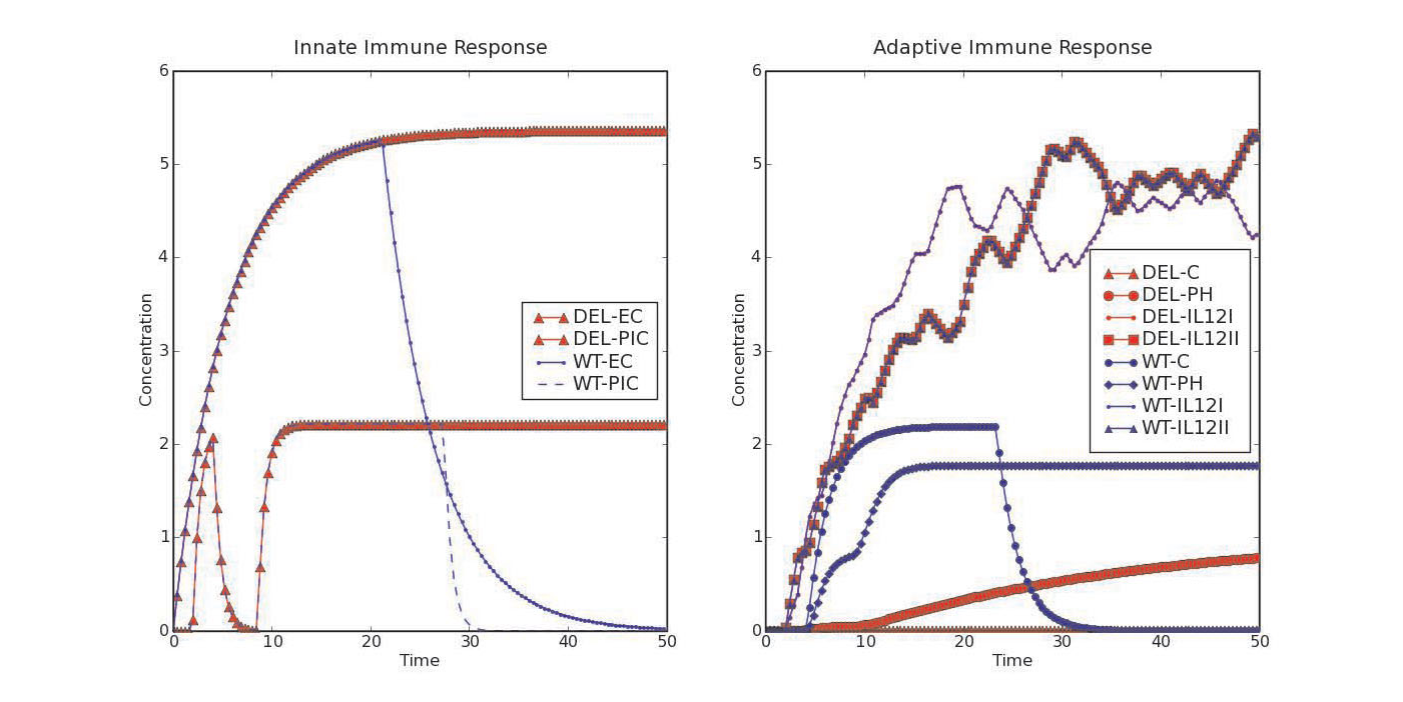
**The time course of (a) innate and (b) adaptive immune response to B.** bronchiseptica is shown by plotting the dynamics of the following representative nodes: EC: Epithelial cells, PIC: pro-inflammatory cytokines, C: Complement, IL12: Interleukin-12 (I and II indicate concentrations in different compartments) and PH: phagocytosis. Colors demonstrate the difference in the behavior of the nodes in case of normal (blue: wild type simulation) and perturbed (red: deletion simulation) host immunity. The perturbation is modeled by turning off the node representing B cells. The figure shows that innate immune responses are active for a longer period in the deletion simulation due to the persistence of bacteria. Plot b shows that complement is activated in normal simulations but not in the deletion simulation. The figure also shows the coupled fluctuations of the concentrations of IL12 in the two compartments. We can also see that the rate of phagocytosis is much slower in the deletion simulation.

Figure 3 shows the time course of components of the innate and adaptive immune response generated by BooleanNet. These results reproduce the outcome of a custom computer code written specifically for simulating host-pathogen interactions (unpublished data). Additionally the software runtime is shorter and it offers a generalized framework which can be used to simulate different systems. BooleanNet also provides extra features including a user friendly way to search in the parameter space and to import parameter files.

We also simulated previously known network perturbations e.g. B cell, T cell deletion and the infections by Type III secretion system (TTSS) defective Bordetella strain. The model could reproduce the persistence of bacteria in the absence of B cells (see Figure 3, DEL) and T cells [27,28]. It can also simulate the earlier clearance of bacteria in Δ*bscN *mutant (TTSS defective strain) infection [29].

## Conclusion

The success of qualitative models in describing specific cellular systems and processes such as flower development [30,31], the yeast cell cycle [32], and Drosophila embryonic development [33-35] indicates that the topology of regulatory networks has a significant role in restricting their dynamical behavior. Our software allows straightforward implementation of qualitative models for systems where the network topology or pathway is at least partially known.

## Authors' contributions

IA programmed the software and wrote the paper. SL performed case study 1. RZ performed case study 2. JT performed case study 3. RA directed research and wrote the paper.

## Competing interests

The author(s) declare that they have no competing interests
